# Low Concentrations of Chlorhexidine Inhibit the Formation and Structural Integrity of Enzyme-Treated Multispecies Oral Biofilms

**DOI:** 10.3389/fmicb.2021.741863

**Published:** 2021-09-28

**Authors:** Kay Andrin Gränicher, Lamprini Karygianni, Thomas Attin, Thomas Thurnheer

**Affiliations:** Clinic of Conservative and Preventive Dentistry, Center of Dental Medicine, University of Zurich, Zurich, Switzerland

**Keywords:** multispecies oral biofilm, chlorhexidine, proteinase K, DNase I, extracellular polymeric substances, confocal laser scanning microscopy

## Abstract

The self-produced matrix of biofilms, consisting of extracellular polymeric substances, plays an important role in biofilm adhesion to surfaces and the structural integrity of biofilms. In dentistry, biofilms cause multiple diseases such as caries, periodontitis, and pulpitis. Disruption of these biofilms adhering to dental hard tissues may pose a major challenge since biofilms show higher tolerance to antimicrobials and antibiotics than planktonic cells. In this study, the effect of low concentrations of chlorhexidine (CHX) on enzyme-treated multispecies oral biofilm was investigated in an *in vitro* model. Six-species biofilms were enzymatically treated by anaerobic growth in a medium containing DNase I and proteinase K. Biofilms were exposed to a low concentration of CHX at defined time points. After 64h, biofilms were either harvested and quantified by cultural analyses or stained for confocal laser scanning microscopy (CLSM) analyses using either Live/Dead kit or different fluorescent dyes. A mixture of YoPro1 and SYTOX^™^ Green, Fluorescent Brightener 28 (Calcofluor), and SYPRO^™^ Ruby Protein Gel Stain was used to stain total DNA, exopolysaccharides, and extracellular proteins, respectively. Extracellular DNA (eDNA) was visualized *via* an indirect immunofluorescence assay (Mouse anti-DNA IgG, Goat anti-Mouse IgG, Streptavidin-Cy3). Overall, the total colony-forming units significantly decreased after combined treatment with a low concentration of CHX and enzymes compared to the group treated with CHX alone (*p*<0.001). These findings also apply to five species individually (*Streptococcus mutans, Streptococcus oralis, Actinomyces oris, Veillonella dispar,* and *Candida albicans*) occurring in the biofilms, with *Fusobacterium nucleatum* being the only exception. Furthermore, CLSM images showed less dense biofilms and a reduction in cell numbers after combined treatment compared to the group without enzymes. The combination of enzymes capable of disturbing the matrix integrity with antimicrobial agents thus appears to be a promising approach for biofilm disruption and killing.

## Introduction

Biofilms are defined as matrix-enclosed bacterial populations adherent to each other and/or to surfaces or interfaces (Costerton et al., 1995). In dentistry, biofilms cause multiple oral diseases, such as periodontitis or caries, which constitute the most prevalent infectious oral diseases and the main causes of tooth loss (Sanz et al., 2017). Initially, the bacterial cells adhere to the pellicle-coated tooth surface *via* different receptor proteins (Kolenbrander et al., 2010). The cells in a biofilm are embedded in a self-produced matrix consisting of extracellular polymeric substances (EPS) such as exopolysaccharides, proteins, extracellular DNA (eDNA), and lipids (Flemming and Wingender, 2010). The functions of a biofilm matrix are diverse and include adhesion to biotic and abiotic surfaces, cohesion, scaffolding, and protection against antimicrobials and dispersal (Flemming, 2016; Flemming et al., 2016). Another key function of the biofilm matrix is to retain nutrients and water to prevent desiccation (Marsh, 2005). In our work, we focused on two EPS, namely, eDNA and proteins, that both serve as matrix components. Many studies show that eDNA plays an important role in structural stability in an early phase of biofilm formation (Whitchurch et al., 2002; Schlafer et al., 2017). The eDNA forms nanofibers connecting cells to the substrate or other cells in the biofilm, thereby mechanically stabilizing the biofilm (Liao et al., 2014). There is evidence that eDNA is released *via* membrane vesicles upon activation of different mechanisms, including cell lysis and active secretion (Jakubovics et al., 2013; Liao et al., 2014). It is also known that bacteria in biofilms are more resistant to antimicrobial agents than in the planktonic phase, which can be attributed to different mechanisms (Flemming et al., 2016). For example, due to its negative charge, eDNA can bind cationic antimicrobials, such as chlorhexidine (CHX; Okshevsky and Meyer, 2015). Another mechanism of biofilm tolerance toward CHX is the upregulation of multidrug efflux pumps, which are membrane proteins containing multiple transmembrane domains that allow for the elimination of antimicrobials and antibiotics from the cytoplasm. These proteins have been found in Gram-positive *Staphylococcus aureus* and in Gram-negative *Pseudomonas aeruginosa* (Cieplik et al., 2019). The diverse functions of matrix proteins have been shown in several studies and include, for example, the binding of eDNA (Kavanaugh et al., 2019) or scaffolding the matrix by forming amyloid fibers (Romero and Kolter, 2014; Erskine et al., 2018).

Using enzymes or other agents for the disruption of the biofilm matrix could be a promising approach to control biofilms in the future (Jakubovics et al., 2013; Kostakioti et al., 2013; Nguyen et al., 2014). This could lead to new treatment approaches, especially in the fields of cariology, periodontology, endodontology, or even prosthodontics, by altering biofilm architecture and thereby making biofilms more susceptible to antimicrobials – even in low concentrations. For example, treatment with DNase I can significantly reduce *P. aeruginosa* and *Streptococcus mutans* biofilm growth (Whitchurch et al., 2002; Liao et al., 2014). Schlafer et al. (2017) showed that DNase I treatment of biofilms grown *in vivo* can reduce the biovolume of biofilms in a statistically significant range. Another study showed that treatment of *S. aureus* biofilms with proteinase K can reduce early microbial adhesion, enhance dispersal of bacterial cells, and increase susceptibility toward different antibiotics (Kumar Shukla and Rao, 2013). In a recently published study, we tested the combined effects of DNase I and proteinase K as examples of “anti-matrix” agents on a multispecies oral biofilm (Karygianni et al., 2020a). In that study, it was shown that the combined application of DNase I and proteinase K had an impact on the reduction in total colony-forming units (CFU) and on the biofilm structure. The aim of the current study was to investigate the effects of CHX on DNase I/proteinase K-treated biofilms, thereby combining antimicrobial agents with “anti-matrix” agents using the “Zurich biofilm model.” Thus, in addition to matrix-degrading enzymes, biofilms were exposed to high (≥0.1%) and low (<0.1%) concentrations of CHX (Serrano et al., 2015). We therefore quantified CFU and visualized the biofilms with the aid of confocal laser scanning microscopy (CLSM) to show alterations in structure and composition in multispecies oral biofilms. To the best of our knowledge, the combined effect of DNase I, proteinase K, and CHX has never been studied so far in an oral *in vitro* multispecies biofilm. The null hypothesis of this study was that there is no difference in microbial growth between enzyme-treated biofilms and untreated biofilms when exposed to high (≥0.1%) and low (<0.1%) concentrations of CHX.

## Materials and Methods

### Formation of Multispecies Supragingival Biofilms

The formation of the biofilm, consisting of six species of microorganisms typically found in supragingival biofilms, was performed *in vitro* as previously described in detail (Shapiro et al., 2002; Thurnheer et al., 2003, 2006, 2014). In brief, *S. mutans* OMZ 918*, Streptococcus oralis* OMZ 607*, Actinomyces oris* OMZ 745*, Veillonella dispar* OMZ 493*, Fusobacterium nucleatum* OMZ 598, *and Candida albicans* OMZ 110 were used for biofilm formation. All strains were maintained on Columbia blood agar (CBA). Prior to the commencement of the biofilm experiments, all strains were transferred into modified fluid universal medium (mFUM; Gmür and Guggenheim, 1983) and anaerobically incubated at 37°C for two cycles of precultures (16 and 8h, respectively). For the production of the inoculum, all strains were adjusted to a defined optical density (OD) at 550nm (OD_550_=1.0) and mixed in equal volumes (1ml).

The experimental biofilms were grown in 24-well polystyrene cell culture plates on sintered hydroxyapatite disks (Ø 9mm; Clarkson Chromatography Products, Inc. South Williamsport, PA 17702, United States) that were preconditioned in 800μl of pasteurized whole unstimulated pooled human saliva (termed “saliva” in the following) from donors of the division of Clinical Oral Microbiology and Immunology at the University of Zurich with their consent and incubated at room temperature for 4h while gently shaking at 95rpm to enable pellicle formation on the disks. The detailed procedure for the collection and processing of the saliva was previously described elsewhere (Guggenheim et al., 2001).

In order to disrupt the biofilm matrix and to prevent biofilm formation, DNase I (from bovine pancreas; Sigma-Aldrich, Buchs, Switzerland), proteinase K (from *Engyodontium album*; Sigma-Aldrich), and chlorhexidine digluconate solution (CHX, Sigma-Aldrich) were used. The experimental setup involved four treatment groups, with each group being divided into two subgroups whose growth medium contained either no enzymes or DNase I+proteinase K (Table 1): group A was treated with 0.05% CHX, group B with 0.1% CHX; group C with 0.2% CHX, and group D was not treated and served as the negative control. The timing of exposure and incubation time as well as enzyme concentrations were optimized in preliminary experiments (data not shown). The growth medium contained 70% saliva and 30% mFUM supplemented with Sørensen’s buffer (pH 7.2). For those biofilms treated with DNase I and proteinase K, a stock solution of each enzyme was added to the media resulting in a final concentration of 0.002mg/ml for DNase I and 0.1mg/ml for proteinase K. The carbohydrate concentration of mFUM was 0.3% glucose (w/v; 0–16h of biofilm cultivation) or 0.15% glucose and 0.15% sucrose(16–64h). To initiate a biofilm experiment, disks were covered with 1.6ml of growth medium and 200μl of inoculum prior to anaerobic incubation at 37°C. The growth medium was changed after 16 and 40h. After 16, 20, and 24h, and after 40, 44, and 48h, the disks were placed in 1ml CHX in the respective concentration for 1min (see treatment groups in Table 1). To remove the non-adherent microorganisms and remaining CHX, the biofilms were washed by consecutively dipping them three times in 2ml saline before putting them back into the medium.

**Table 1:** Arrangement of the biofilms into eight different treatment groups.

Group	Growth medium supplement	Treatment^a^
A1	No enzymes	0.05% CHX
A2	0.002mg/ml DNase I plus 0.1mg/ml proteinase K	0.05% CHX
B1	No enzymes	0.1% CHX
B2	0.002mg/ml DNase I plus 0.1mg/ml proteinase K	0.1% CHX
C1	No enzymes	0.2% CHX
C2	0.002mg/ml DNase I plus 0.1mg/ml proteinase K	0.2% CHX
D1	no enzymes	–
D2	0.002mg/ml DNase I plus 0.1mg/ml proteinase K	–

a *CHX treatment was performed at six different time points (16, 20, 24, 40, 44, 48h) for 1min each.*

After 64h of biofilm growth, the biofilms were washed as described and harvested for culture analyses or proceeded to staining and CLSM (see below).

### Culture Analyses and CFU Counting

For culture analyses, the disks were placed in a tube and vortexed vigorously for 1min in 1ml of 0.9% NaCl to harvest the adherent biofilms. After vortexing, the harvested biofilms were sonicated at 30W for 5s (Sonifier B-12, Branson Ultrasonic, Urdorf, Switzerland) to ensure that the microorganisms were dispersed. The resulting bacterial suspensions were serially diluted in 0.9% NaCl. Of each dilution, 50μl aliquots were plated on CBA (Oxoid Ltd., Basingstoke, United Kingdom) supplemented with 5% whole human blood to estimate total CFUs. Selective agars were used to determine the CFUs of the species in the biofilm as described earlier (Guggenheim et al., 2001; Klinke et al., 2011). In brief, CBA plates were used to obtain total bacterial counts and to enumerate *A. oris* and *V. dispar*. Differential counting of *S. mutans* and *S. oralis* was performed using Mitis Salivarius Agar (Difco Laboratories, Inc., Detroit, MI) supplemented with 0.001% (w/v) sodium tellurite, whereas selective growth of *F. nucleatum* was achieved with Fastidious Anaerobe Agar (Chemie Brunschwig, Basel, Switzerland). Lastly, BIGGY Agar (BBL, Becton Dickinson, Allschwil, Switzerland) was used to enumerate *C. albicans.* Agar plates were incubated, either anaerobically for CBA and Fastidious Anaerobe Agar plates or aerobically plus 10% CO_2_ for BIGGY and Mitis agar plates, at 37°C for 72h. Species were identified through observation of colony morphology using a stereo loupe. Finally, phase-contrast microscopic identification of cells from selected colonies was performed.

### Staining of the Biofilm for CLSM Analysis

Two different staining methods were used to visualize the biofilm. The first involved staining the total bacteria and various matrix components such as extracellular DNA (eDNA), extracellular polysaccharides (EPS), and extracellular proteins, whereas the second aimed to visualize either intact or ruptured cell membranes using the LIVE/DEAD^™^ BacLight^™^ Bacterial Viability Kit.

Prior to staining the total bacteria and matrix components, the biofilms were fixed in 1ml of 4% paraformaldehyde + RNase inhibitor (RNAi; Sigma-Aldrich) for 2h at 4–8°C. After fixation, disks were washed in 500μl 0.9% NaCl + RNAi and dabbed off on a paper towel. Total DNA was stained with a mixture of 3μM YoPro 1 iodide (Thermo Fisher Scientific, Basel, Switzerland) and 15μM SYTOX^™^ Green (Thermo Fisher Scientific) in nanopure water for 30min in the dark at room temperature. Extracellular polysaccharides were stained by incubating biofilms with Fluorescent Brightener 28 (Calcofluor; Sigma-Aldrich; 10μg/ml solution in 10mM sodium phosphate, pH 7.5) for 30min in the dark at room temperature. Extracellular DNA (eDNA) was visualized using an indirect immunofluorescence assay (Mouse anti-DNA IgG (Sigma-Aldrich), Goat anti-Mouse IgG (Thermo Fisher Scientific), and Streptavidin-Cy3 (GeneTex, Luzern, Switzerland) according to the manufacturer’s recommendations, while extracellular proteins were stained with SYPRO^™^ Ruby Protein Gel Stain (Thermo Fisher Scientific) according to the manufacturer’s protocols.

Biofilms stained with the LIVE/DEAD^™^ BacLight^™^ Bacterial Viability Kit (L7007; Thermo Fisher Scientific) were not fixed prior to staining. The biofilms were dyed with SYTO9 and propidium iodide following the manufacturer’s instructions.

After staining, the samples were washed and embedded upside down on chamber slides in 50μl of Mowiol (Thurnheer et al., 2003).

### Confocal Laser Scanning Microscopy

CLSM was conducted using a Leica TCS SP5 microscope (Leica Microsystems, Wetzlar, Germany) provided by the Center for Microscopy and Image Analysis of the University of Zurich. For the imaging of the biofilms on hydroxyapatite, the slightly modified procedure, as described previously (Zehnder et al., 2017), was performed. In brief, the lasers used were a UV laser at 405nm excitation, an Argon laser at 488nm excitation, a DPSS diode laser at 561nm, and a Helium-Neon laser at 633nm excitation. Furthermore, filters were adjusted at 420–470nm to detect Calcofluor, at 495–555nm for YoPro1/SYTOX^™^ Green and SYTO9, at 575–620nm for Cy3 and propidium iodide, and at 650–720nm for SYPRO^™^ Ruby Protein Gel Stain. To visualize total bacteria and matrix components, an x100 oil immersion objective (numerical aperture 1.4) was used, and biofilms were scanned sequentially in steps of 0.5μm thickness. LIVE/DEAD-stained biofilms were examined using an x63 oil immersion objective (numerical aperture 1.4), and biofilms were scanned sequentially in steps of 0.29μm thickness. Finally, the images were processed using Imaris 64 8.4.1 (Bitplane, Zurich, Switzerland).

### Statistical Analysis

All experiments were conducted twice in triplicate, resulting in *n*=6 samples in each group. Two-way ANOVA was used to analyze the difference in bacterial cells per biofilm between the control group (standard six-species biofilm) and the different treatments with either CHX in different concentrations alone or in combination with DNase I/proteinase K. Tukey’s multiple comparisons test was used for correction. Missing values were ascribed to the lowest detection limit value of the assay to allow for logarithmic transformation. Statistics were implemented using GraphPad Prism software (version 7; La Jolla, CA, United States) to compare the species’ total cell counts within the different biofilm formations. The significance level was set at *p*<0.05.

## Results

### Combined Treatment With CHX and DNase I/Proteinase K Reduces Biofilm Formation

**Figure 1:**
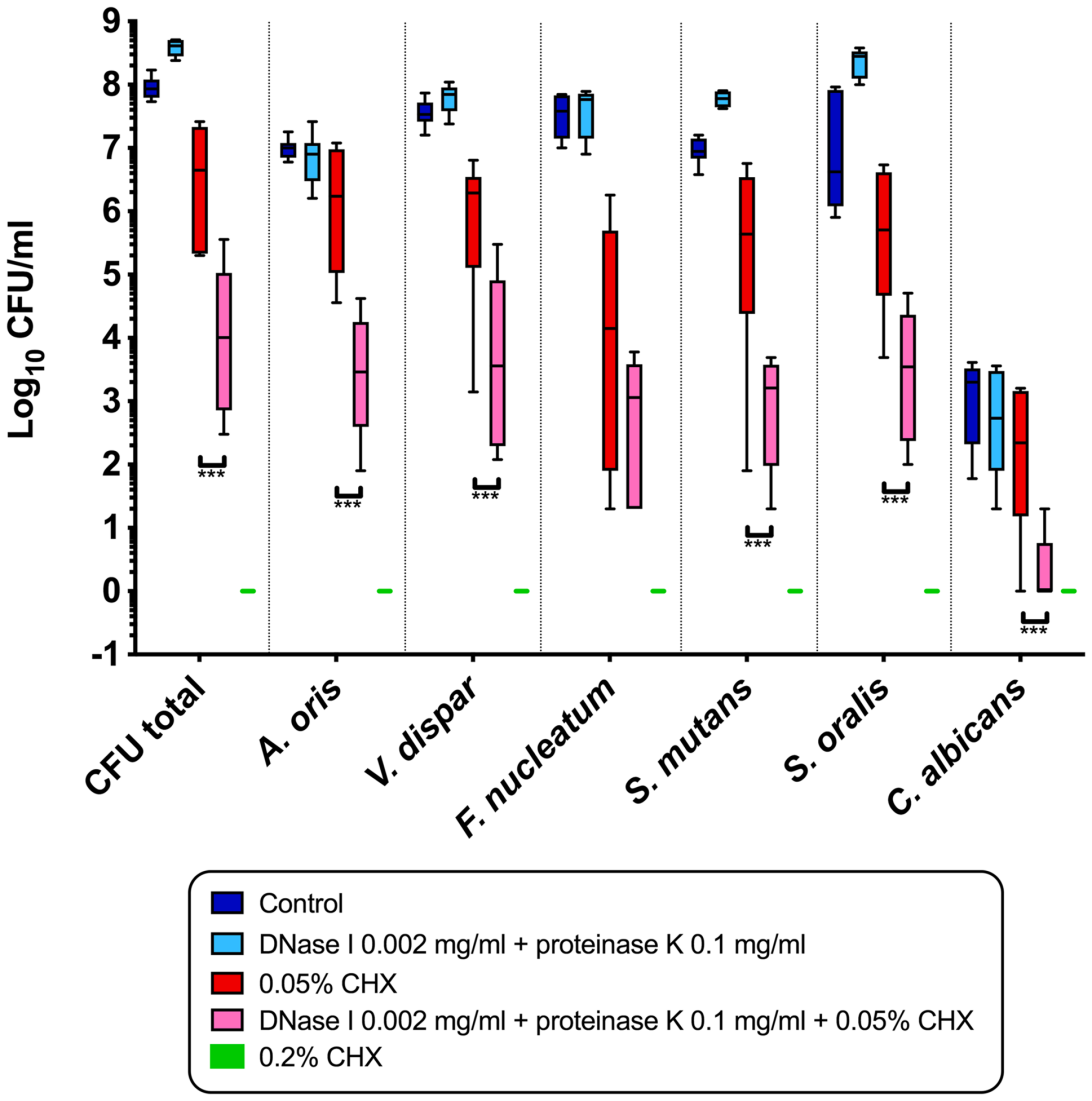
Box plots illustrating the colony-forming units (CFUs) of six-species oral biofilms after simultaneous exposure to 0.002mg/ml DNase I+0.1mg/ml proteinase K (light blue), to 0.002mg/ml DNase I+0.1mg/ml proteinase K+0.05% chlorhexidine (CHX; pink), to 0.05% CHX (red), and to 0.2% CHX (positive control; green). Untreated biofilms were tested as a negative control (dark blue). Data for biofilms treated with proteinase K and DNase I alone were taken from the study of Karygianni et al. (2020a), following the same protocol of biofilm formation. The statistical significance level is indicated with asterisks (^***^ *p*<0.001).

Figure 1 shows the combined effect of CHX and enzymatic treatment with proteinase K and DNase I on the log_10_ CFU counts of supragingival biofilms consisting of six species grown for 64h *in vitro*. The use of 0.05% CHX together with 0.002mg/ml DNase I and 0.1mg/ml proteinase K resulted in a significant decrease in total microbial counts (mean, 3.88±1.09 log_10_ CFU; *p*<0.0001) compared to those of biofilms treated with 0.05% CHX alone (mean, 6.44±0.93 log_10_ CFU). The same effect was measured for five individual microbial species (log_10_ CFU and *p*-values listed in Table 2). The only exception was *F. nucleatum* (mean, 2.58±1.11log_10_ CFU, *p*=0.208), for which no significant reduction compared to the 0.05% CHX-treated biofilm (mean, 3.51±1.93 log_10_ CFU) was observed. Compared to the control (7.95±0.17 log_10_ CFU, *p*<0.0001), a significant reduction in *F. nucleatum* after biofilm treatment with 0.05% CHX alone was achieved.

**Table 2:** Mean log_10_ CFU counts of the whole biofilm (total CFU) and the six microorganisms used in the biofilm model when treated with 0.05% CHX compared to the combined treatment with enzymes plus 0.05% CHX alone (*n=6).*

Microorganism	0.05% CHX	0.002mg/ml DNase I + 0.1mg/ml proteinase K+0.05% CHX	
	Mean log_10_ CFU±SD	Mean log_10_ CFU±SD	*p*
Total CFU	6.44 ± 0.93	3.88 ± 1.09	<0.0001
*A. oris*	6.01 ± 1.01	3.35 ± 0.96	<0.0001
*V. dispar*	5.69 ± 1.35	3.46 ± 1.23	<0.0001
*F. nucleatum*	3.51 ± 1.93	2.58 ± 1.11	0.2080
*S. mutans*	5.05 ± 2.78	2.74 ± 0.97	<0.0001
*S. oralis*	5.44 ± 1.19	3.39 ± 1.02	<0.0001
*C. albicans*	1.98 ± 1.25	0.22 ± 0.53	0.0008

Biofilms treated with 0.2% CHX were used as a positive control, and no microbial growth was detected in this group. Data from the biofilms treated with 0.1% CHX are not shown in Figure 1 because there was no significant difference between this group and the positive control.

### CLSM Images Show Loss of Density and Disturbance of Structural Integrity of the Biofilms After Combined Treatment With CHX and DNase I/Proteinase K

**Figure 2:**
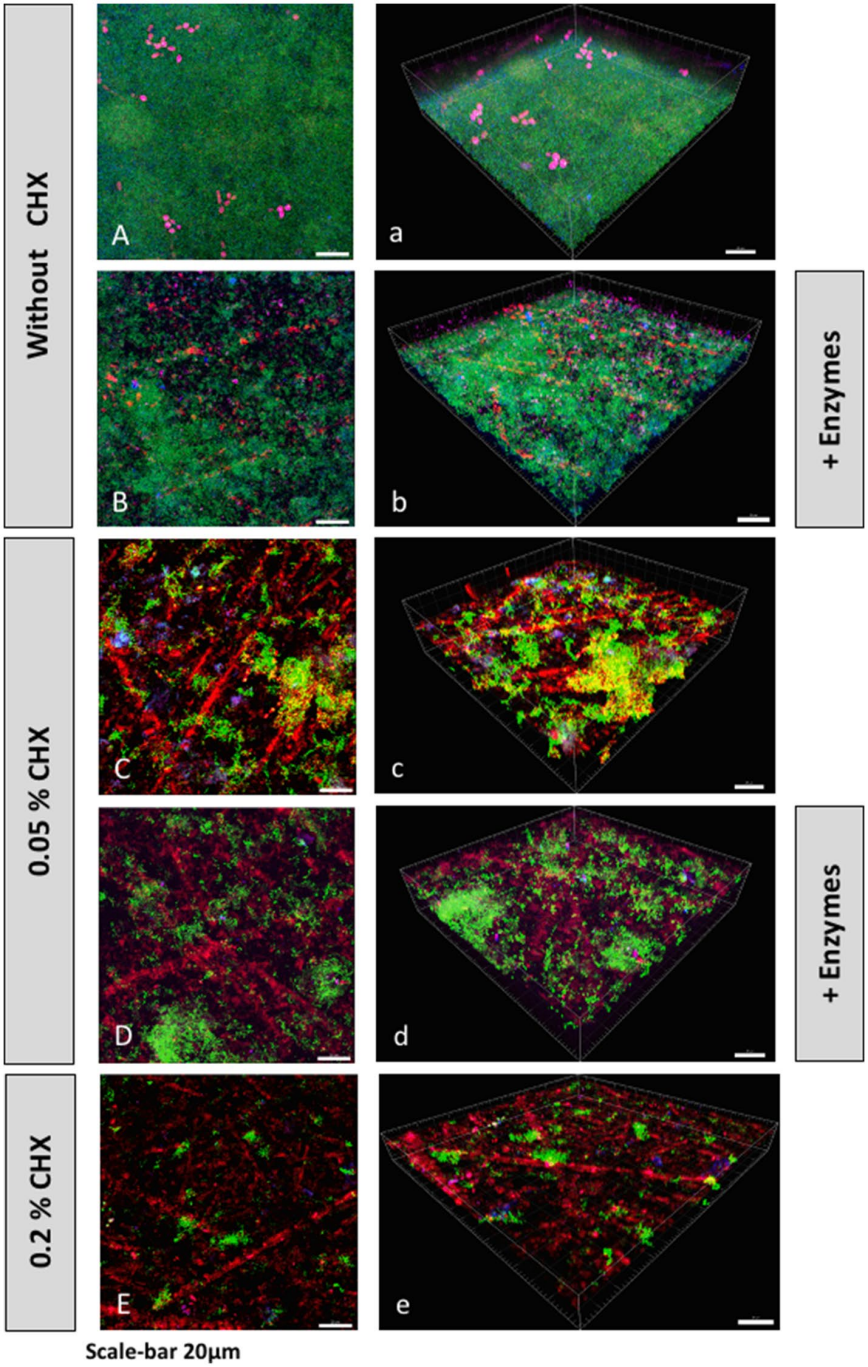
Confocal laser scanning microscopy (CLSM) Z-stack maximum projections **(A–E)** and 3D reconstructions **(a–e)** of biofilms grown on HA disks after treatment with or without CHX and/or DNase I+proteinase K. Total DNA (green) was stained with a mixture of YoPro 1 and SYTOX^™^ Green. Extracellular polysaccharides (blue) were stained with Calcofluor. Extracellular DNA (eDNA; red) was stained with Cy3-streptavidin labeled anti-DNA-antibody, and extracellular proteins (purple) were stained with SYPRO^™^ Ruby.

Representative CLSM images of fluorescently stained biofilms after enzymatic, antimicrobial (CHX), or combined treatment are shown in Figure 2. Panels indicated with majuscules (A–E) are maximal projections of Z stacks, while those indicated with minuscules (a–e) are 3D reconstructions of the respective Z stacks. Total DNA appears green due to staining with a mixture of YoPro 1 iodide and SYTOX^™^ Green, whereas extracellular polysaccharides appear blue due to staining with Calcofluor. Extracellular DNA (eDNA) was stained with Cy3-streptavidin labeled anti-DNA-antibody and appears red, while extracellular proteins were stained purple with SYPRO^™^ Ruby. Panels 2A/2a represent the untreated biofilms. They show dense, intact biofilms with some protein clusters. Enzymatically treated biofilms (0.002mg/ml DNase I plus 0.1mg/ml proteinase K) are shown in panels 2B/2b. From the results, it is apparent that the treatment with both enzymes (DNase I, proteinase K) resulted in less dense biofilm masses with less visible protein clusters compared to the untreated biofilms. Panels 2C/2c depict biofilms treated with 0.5% CHX, and panels 2D/2d show biofilms treated with 0.5% CHX, 0.002mg/ml DNase I and 0.1mg/ml proteinase K. The density of the biofilms appears to decrease after treatment with the lowest concentration of CHX (0.05%) and even more after combined treatment with the lowest concentrations of CHX and DNase I/proteinase K. Interestingly, the number of extracellular polysaccharides appears to decrease as the density of cells consecutively decreases in panels 2B-D/2b-2d, respectively, although we did not use any polysaccharide digesting enzymes in the experiments. Panels 2E/2e represent the biofilms treated with 0.2% CHX (positive control), which show practically no more cells and proteins.

### Live/Dead Staining Shows a Reduction in Cell Numbers After Combined Treatment With CHX and DNase I/Proteinase K

**Figure 3:**
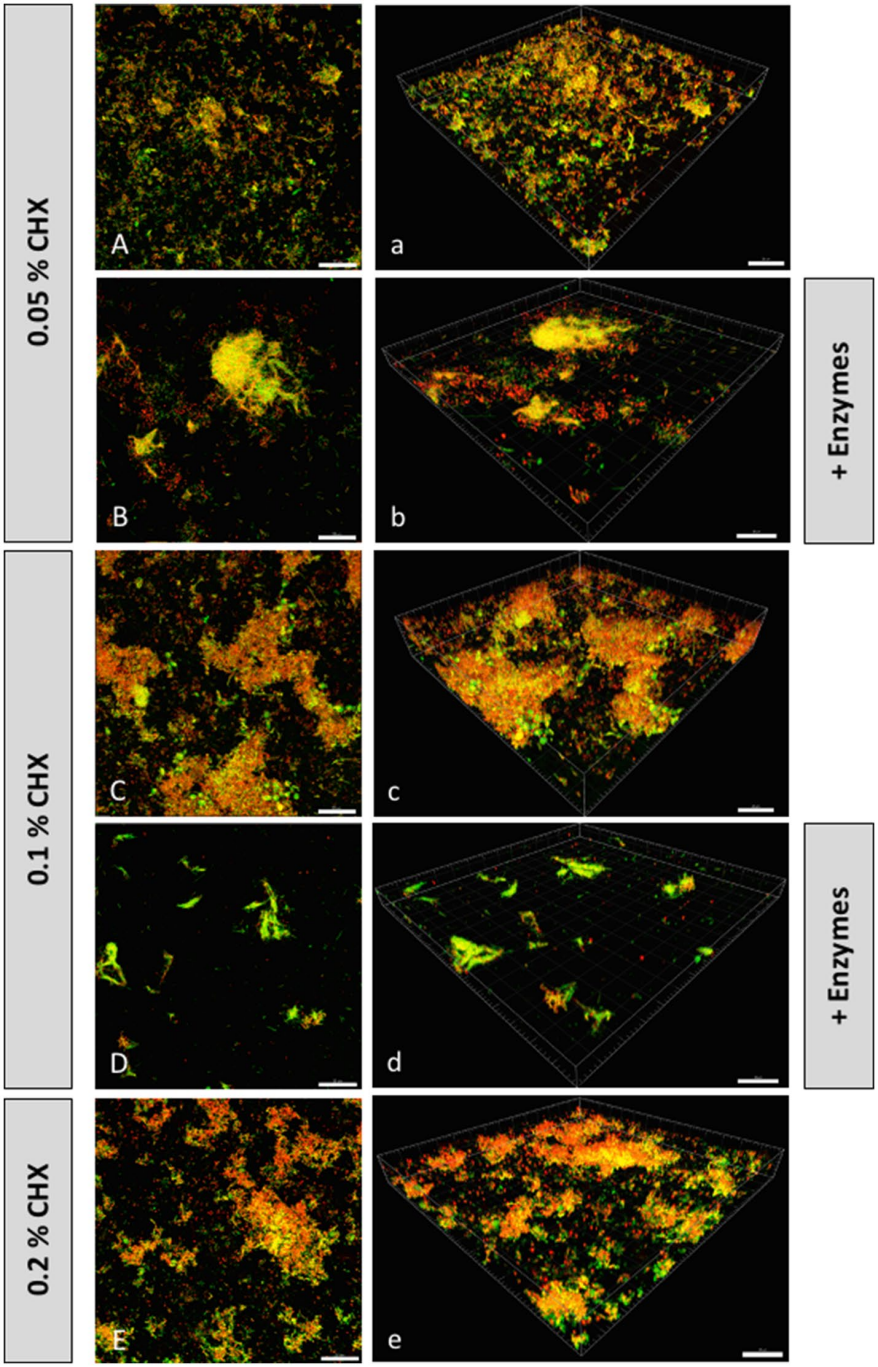
Confocal laser scanning microscopy (CLSM) Z-stack maximum projections **(A–E)** and 3D reconstructions **(a–e)** of biofilms grown on HA disks after treatment with or without CHX and/or DNase I+proteinase K. Biofilms were stained with the LIVE/DEAD BacLight Bacterial Viability Kit to visualize the relative viability of bacterial populations according to the membrane integrity of the cell. SYTO 9 can penetrate cells with both intact and compromised membranes (green), while propidium iodide only penetrates cells with damaged membranes (red). Scale-bar: 20µm.

Figure 3 shows representative CLSM images of biofilms stained with the Live/Dead BacLight Bacterial Viability Kit to visualize the relative viability of bacterial populations according to the membrane integrity of the cells. The dye SYTO 9 can penetrate cells with both intact and compromised membranes, staining them green, while propidium iodide (PI) only penetrates cells with damaged membranes, staining them red. Panels indicated with majuscules (A–E) are maximal projections of Z stacks, while those indicated with minuscules (a–e) are 3D reconstructions of the respective Z stacks. Panels 3A/3a represent biofilms treated with 0.5% CHX alone, while panels 3B/3b represent biofilms treated with a combination of 0.5% CHX, 0.002mg/ml DNase I and 0.1mg/ml proteinase K. There is an evident reduction in cell density after the combined treatment, with only a few undamaged cells being visible. Similar results are shown in panels 3C/3c when compared to panels 3D/3d. The biofilms in 3C/3c were treated with 0.1% CHX, while biofilms in panels 3D/3d were treated with the same concentration of CHX (0.1%) in addition to DNase I and proteinase K. Panels 3D/3d show only a few cell aggregates, as the density of bacterial cells seems to decrease with the combined enzymatic and antimicrobial treatment. As the CFU counts were already below detection level, the boxplots for these two treatment groups are not shown in Figure 1. Panels 3E/3e show biofilms in the positive control group (0.2% CHX). Here, some adherent bacterial cells are visible, although most of them appear to have membrane defects. This finding is confirmed by the CFU counts for the positive control, which were also below the detection level.

## Discussion

In a previous study (Karygianni et al., 2020a) using the same biofilm model, a low but significant effect of 0.002mg/ml DNase I and 0.1mg/ml proteinase K on total CFU counts of multispecies supragingival biofilms was shown. However, the same enzymatic treatment had a positive effect on the growth of *S. mutans* and a mild, but significant, increase in *S. oralis* growth when compared to the untreated group (negative control). In the present study, we went further by aiming to demonstrate for the first time – to our knowledge – the combined effect of different concentrations of CHX (0.05, 0.1, and 0.2%) with the enzyme DNase I (0.002mg/ml) and proteinase K (0.1mg/ml) on a six-species *in vitro* biofilm, the so-called “Zurich biofilm model.” The application of EPS matrix-digesting enzymes in combination with antimicrobial agents seems to be a promising approach to control biofilm activity (Koo et al., 2017). Hence, the objective of our experiments was to mechanically destabilize the biofilms with the applied enzymes and simultaneously kill the microbes by applying low concentrations of CHX. The combined treatment with DNase I/proteinase K and the lowest concentration of CHX (0.05%) tested led to a significant reduction in total microbial counts and CFU counts of five out of the six species compared to the biofilms treated with 0.05% CHX alone. The only exception was observed in the species *F. nucleatum,* which was already significantly inhibited after CHX (0.05%) treatment alone, but not after the combined treatment with both DNase I/proteinase K and 0.05% CHX.

Confocal microscopy (CLSM) images showed that combined treatment resulted in less dense biofilms, even after treatment with a low CHX concentration of 0.05%. CLSM images are a useful tool to visualize the extracellular matrix of biofilms because they can depict the matrix in a fully hydrated state (Schlafer and Meyer, 2017). Overall, it can be stated that the combined enzymatic and antimicrobial treatment resulted in a reduction of microbial counts and a drastic alteration of the structural integrity within the examined biofilms. In contrast, Ali Mohammed et al. (2013) found no significant effect of proteinase K or DNase I on the formation and dispersion of biofilms consisting of *F. nucleatum* and *Porphyromonas gingivalis* grown in a static biofilm model in polystyrene microtiter plates, although they used much higher concentrations of each enzyme (0.125, 0.25, 0.5 and 1mg/ml).

It is well established that biofilms are more resistant to antibiotics, and antimicrobial agents such as CHX, than planktonic cells (Flemming and Wingender, 2010; Xiao et al., 2012). It appears that ionic interactions hinder the positively charged CHX from diffusing through the negatively charged EPS matrix (Hope and Wilson, 2004). For example, Guiot et al. (2002) showed that *Lactobacillus lactis* and *Stenotrophonas maltophilia* biofilms form a barrier to the diffusion of cationic particles. In another study, Xiao et al. (2012) examined the effect of 0.12% CHX on multispecies biofilms formed with *gtfBC* null mutant strains of *S. mutans,* resulting in rapid killing of these biofilms. The genes *gtfB* and *gtfC* code for glucosyltransferases B or C, respectively (GTF B or GTF C), while the former produces water-insoluble glucans and the latter produces both water-soluble and insoluble glucans (Aoki et al., 1986; Hanada and Kuramitsu, 1988). In the present study, the extracellular polysaccharides were not a direct target of the treatment, but by reducing a large part of the biofilm, extracellular polysaccharides and EPS, in general, are also eliminated. This is particularly evident in Figures 2B–D/b–d. The number of adherent microbial cells decreases because of the treatment with DNase I/proteinase K, which consequently leads to a decreased production of extracellular polysaccharides. The diffusion of CHX into the biofilms is thus facilitated, resulting in the elimination of the remaining microbes. The synergistic effect between CHX in low concentrations (0.5%) and DNase I/proteinase K is indicated in Figure 1, which shows the significant reduction in the total CFU counts and the CFU counts of *A. oris*, *V. dispar*, *S. mutans*, *S. oralis,* and *C. albicans* compared to the treatment with 0.5% CHX alone. These findings indicate that the EPS of a biofilm matrix plays an important role in protecting the biofilm against antimicrobial agents, making it an interesting target for antibiofilm therapy (Karygianni et al., 2020b). In another approach for developing a combined antibiofilm therapy, the susceptibility to four different antibiotics of biofilms formed by *Streptococcus agalactiae, Streptococcus pyogenes,* and *S. oralis* grown on porous sintered glass beads was examined by Gonzalez Moreno et al. (2017). It was shown that pre-treatment of biofilms with proteinase K increased their susceptibility to lower concentrations of the tested antibiotics compared to biofilms treated with antibiotics alone. Previously, it was shown that proteinase K can change the composition of the EPS matrix of *S. aureus* biofilms, particularly by decreasing the number of proteins, and even the amount of eDNA (Shukla and Rao, 2017). Furthermore, proteinase K can disperse established biofilms of *Listeria monocytogenes* by attacking proteins of the matrix used to adhere to a surface (Nguyen and Burrows, 2014). On the other hand, both bovine DNase I and recombinant human DNase I (rhDNase I, dornase alfa) were shown to inhibit biofilm formation of *S. aureus* and *Staphylococcus epidermidis,* disperse preformed biofilms of *S. aureus,* and increase their sensitivity to biocide killing *in vitro* (Izano et al., 2008; Kaplan et al., 2012)*.* Nguyen and Burrows (2014) showed that DNase I can reduce the biofilms of *L. monocytogenes* when present during its formation*.* Bovine DNase I was also able to reduce the formation of *S. mutans* biofilms grown on HA disks by more than 1 log CFU (Liao et al., 2014). Furthermore, rhDNase I can be used clinically in patients with cystic fibrosis to digest neutrophile-derived DNA in order to liquefy their sputum and thereby enhance its clearance (Shak et al., 1990; Suri, 2005; Manzenreiter et al., 2012). Moreover, a single center clinical study tested the use of nebulized dornase alfa together with albuterol (a bronchodilator agent) on five mechanically ventilated patients diagnosed with COVID-19, resulting in reduced oxygen requirement. The patients were all successfully extubated afterward and could be discharged from the hospital (Weber et al., 2020).

The combined use of proteinase K and CHX was already shown to significantly reduce the viable counts of bacteria in a multispecies endodontic biofilm model using different irrigation protocols for root canals when compared to the control groups that were either untreated or treated with saline (Niazi et al., 2015).

Using the outlined methodology, we demonstrated that the combination of matrix-digesting enzymes with antimicrobial agents is a promising approach for biofilm disruption and killing and the null hypothesis of the research was thus disproved.

A wide application of the presented approach is conceivable, especially in the field of dentistry, where for example in daily hygiene procedures for removable prosthesis, a combination of enzymatic and biocidal treatment would be interesting. Some studies showed that a combination of chemical methods and brushing to clean dentures is more effective than brushing alone (Baba et al., 2018; Papadiochou and Polyzois, 2018). It would be interesting to see how the treatment approach to reduce biofilms described here would affect denture materials and whether other biocidal compounds, such as sodium hypochlorite, have the same or an even better effect. These aspects need to be investigated in further trials. Another potential use of enzymes for biofilm disruption in the field of dentistry was described by Niazi et al. (2014) when they investigated the effects of enzymatic irrigation of an endodontic biofilm.

Further experiments are also required to examine the clinical applicability of the approach we developed, especially regarding whether there are any adverse effects when the enzymes are administered intraorally.

## Data Availability Statement

The raw data supporting the conclusions of this article will be made available by the authors, without undue reservation.

## Author Contributions

KG conducted the experiments, analyzed the data, and wrote the manuscript. TA critically reviewed the manuscript. LK and TT conceived the idea for this manuscript, were involved in the data analysis, and critically reviewed the manuscript. All authors read and approved the final manuscript.

## Funding

This study was supported by institutional funds from the University of Zurich.

## Conflict of Interest

The authors declare that the research was conducted in the absence of any commercial or financial relationships that could be construed as a potential conflict of interest.

## Publisher’s Note

All claims expressed in this article are solely those of the authors and do not necessarily represent those of their affiliated organizations, or those of the publisher, the editors and the reviewers. Any product that may be evaluated in this article, or claim that may be made by its manufacturer, is not guaranteed or endorsed by the publisher.
